# Oral health knowledge, behavior, and barriers to dental care of adult Jordanians adorning oral and/or perioral piercings-a cross-section study

**DOI:** 10.3389/froh.2025.1573786

**Published:** 2025-06-19

**Authors:** Sabha Mahmoud Alshatrat, AbdelRahman Murtada Ramadan, Hanan M. Hammouri, Yousef Saleh Khader, Isra Abdulkarim Al-Bakri, Tamadur Mahmoud Falah, Abedelmalek Kalefh Tabnjh

**Affiliations:** ^1^ Department of Applied Dental Sciences, Jordan University of Science and Technology, Irbid, Jordan; ^2^ Council of Periodontology, Sudan Medical Specialization Board, Khartoum, Sudan; ^3^ Department of Mathematics and Statistics, Faculty of Arts and Science, Jordan University of Science and Technology, Irbid, Jordan; ^4^ Department of Community Medicine, Jordan University of Science and Technology, Irbid, Jordan; ^5^ Department of Cariology, Institute of Odontology, The Sahlgrenska Academy, University of Gothenburg, Gothenburg, Sweden; ^6^ Dental Research Unit, Center for Global Health Research, Saveetha Medical College and Hospital, Saveetha Institute of Medical and Technical Sciences, Chennai, India

**Keywords:** behavior, barriers, complications, Jordanians, knowledge, oral health, piercing

## Abstract

**Background:**

The global prevalence of oral piercings is increasing, and there are mounting concerns about complications associated with oral and/or perioral piercings. Providing precautionary advice about piercing complications is important.

**Aims:**

to determine the oral health knowledge, behavior, and barriers to dental care for oral and/or perioral piercings in adult Jordanians.

**Methods:**

A web-based, anonymous, self-administered closed-end questionnaire was distributed across Jordan. It included questions regarding oral health knowledge, behavior, and barriers to dental care.

**Results:**

About (81.5%) liked how it looked. Most participants (49%) reported no complications, while 35% reported pain. The beauty parlors placed 76% of piercings and were also the source of help in case of complications. Most common barriers to seeking regular care were the perception that health professionals would refuse to treat them and the lack of confidence in the health professionals (90%) to treat the complications. Most participants (47%) brushed their teeth at least twice a day, and 68% spent 1–2 min brushing. Most participants (86%) knew that sugars and sweets caused dental caries. Also, (73%) believed bleeding gums was abnormal.

**Conclusion:**

This study suggests that adult Jordanians primarily choose piercings for aesthetic reason, with beauty parlors being the preferred place for both piercings and assistance in the event of complications. The lack of trust in healthcare professionals, with the believe that experts may refuse treatment were the reasons for participants avoided seeking regular dental care, which might increase risk of periodontal and gingival diseases.

## Background

Several civilizations have carried out body modifications, including tattooing and body piercing, over time in diverse geographic areas and with well-defined civilizing and societal implications (1).

Piercings are typically located intraorally on the tongue or periorally on the lips, cheeks, frenulum attachments, or a combination of these sites. There are two types of oral piercings: oral piercing, which involves the ornament's two ends being inserted into the oral cavity, and extra-oral piercing, which involves one end being inserted into the oral cavity and the other piercing the skin, such as a lip piercing (2). The most commonly used jewelry for oral piercings are barbells of various sizes, which have been reported in various oral sites into the various sites without anesthesia using a needle of a similar diameter to the barbells. A tongue piercing is usually observed anterior to the lingual frenum in the middle of the tongue, and once the ornament is in place, it is continuously worn to prevent the site from closing (3).

The acceptance of piercing has grown in contemporary years and has been associated with “self-expression.” Nevertheless, the rising demand from young adults and teenagers for oral and/or perioral piercings has created interest within the medical and dental profession concerning the risk presented to the individual by this procedure (1). The risk of infection control for blood-borne infections is underscored by the fact that piercing is typically performed in a tattoo or body piercing parlor, where the individuals conducting the process have not received adequate sterilizing or cross-infection control instruction (4).

A tongue piercing is a common procedure involving the tongue's perforation with a big needle, resulting in swelling and stiffness. The healing process typically takes approximately 4 weeks. The most significant complications are also included in tongue piercing (5).

A piercing on the frenulum of the tongue, a small mucous membrane fold stretching from the midline of the tongue's ventral surface to the mouth's floor, also takes almost 4 weeks to heal but is usually easily rejected by the body (6). Oral piercings have been linked to a variety of oral health disorders, such as dental caries, periodontal issues, tooth chipping, and bleeding. The more time the piercings are worn, the more likely these complications occur (5–7).

Although these potential issues exist, there is a lack of understanding among individuals with oral piercings and those who perform the procedure (8, 9). The dental profession is uniquely positioned to detect oral and dental problems early and provide evidence about preventing and maintaining oral piercings (7). The need for education programs targeted at young adults is widely recommended in the literature (8–10).

Due to the global prevalence of oral piercing, young adults and teenagers need to be educated about piercing to fathom the extent and dangers of oral piercing. A comprehensive literature review revealed no research had been conducted in Jordan to identify the knowledge, behavior, and barriers associated with oral and perioral piercings. Consequently, this study aims to assess oral health knowledge, behavior, and barriers to dental care for adult Jordanians with perioral and/or oral piercings.

## Methods

### Study design

An observational cross-sectional web-based study was conducted to determine the oral health knowledge, behavior, and barriers to dental care for oral and/or perioral piercings in young adult Jordanians.

### Study duration

After consulting with a statistician, the target sample size was estimated using power analyses. Based on a power analysis using an average proportion of 34.5% [as reported by Covello et al. (7)], a precision level of 10%, and a significance level of 0.05, the calculated sample size was 87 participants. A total of 90 participants were recruited. A confidence interval (CI) of 95%, a standard deviation of 0.5, and a margin of error of 5% were employed. The study participants were recruited using a convenience sample.

### Selection criteria

#### Inclusion criteria

Jordanians working in Jordan and willing to participate in the study wore oral and/or perioral piercings.

#### Exclusion criteria

The study excluded individuals who declined participation and those who did not wear oral and/or perioral piercings.

### Assessment procedure

A web-based anonymous self-administered closed-end questionnaire was generated through the Google Forms application for data collection. The generated Google forms were distributed to participants via social media applications like Facebook and WhatsApp and through the various websites of Jordanian social networking, beauty parlor, and Jordanian schools/universities after obtaining their permission to use their websites. The questionnaire was developed for this study, except regarding the reason(s) for getting (having) a piercing. The question was previously published in Gold et al. (11). The questionnaire form was prepared in English and then translated into Arabic. An Arabic version was distributed to the participants. Then, the Arabic version was retranslated to English by bilingual specialists to certify uniformity.

The questionnaire was submitted to an expert panel of oral health professionals, who were requested to respond and provide input on its content, completion time, clarity, and structure. The final version was provided in Arabic to the participants once all the necessary revisions were done. The Average Congruency Percentage (ACP) score of 92% demonstrated the questionnaire's validity. The questionnaire was administered to the same participants (*n* = 10) on two different dates, establishing test-retest reliability. The questionnaire's internal reliability was evaluated using Cronbach's alpha. Indicating that the items had satisfactory internal consistency, Cronbach's alpha coefficient was 0.75. The questionnaire was pilot-tested with ten volunteers, but the results were not included in the final data collection.

The questionnaire was divided into four sections. Section 1: Demographics comprised four items (age, gender, education level, and marital status). Section 2: Questions for individuals with oral and/or perioral piercing comprised ten items. Section 3: Four items comprised oral health behavior for individuals with oral and/or perioral piercing (s). Section 4: Ten items comprised the oral health information for those with oral and/or perioral piercings. Participation in the study was voluntary, and the participants were encouraged to participate by explaining the extent of anonymity and the importance of the research to people's health. Complete confidentiality of the collected data was secured in the statistician's password-protected laptop. The data from the received questionnaires were first downloaded into an Excel spreadsheet before being transformed into IBM-SPSS. The received data was anonymized and de-identified prior to analysis. Completing the questionnaires was considered a proxy for consent to participate in the study.

Questions regarding knowledge and behavior were awarded a score of “one” for a true answer and zero for false and don't know answers. An individual score of less than 50% (1–7 score), 51%–75% (8–10 score), and 76%–100% (11–14 score) were considered poor, moderate, and good, respectively.

### Data analysis

Data were collected and analyzed using the IBM SPSS Statistics for Windows, Version 29.0. All the statistical methods used were two-tailed with an alpha level of 0.05, and a *p*-value <0.05 was considered significant. A Chi-square test and contingency-table analysis were performed on the data.

## Results

A total of 90 participants completed the questionnaire. Among the participants, the majority (39%) were between the ages of 21 and 25, while the least (8%) were under 15. The study was primarily conducted with female participants (98%), and the majority of the participants (52%) held a bachelor's degree. Furthermore, the majority of respondents (75%) were single.

More than 2 years was the response of the majority (38%) to the question, “How long have you been putting piercings?” In response to the question, “Who is responsible for placing your oral and/or perioral piercings?” The majority of participants (76%) reported that their piercings were placed in a beauty parlor, while roughly (15%) claimed that a nurse performed the procedure. None of the interviewees asserted that a dentist was involved in the placement of piercings. Nearly seven % of respondents asserted that a physician inserted their piercings. An additional eight % asserted they were “other” (a goldsmith, a pharmacist, or themselves).

The majority of participants (71%) responded “Yes” to the question, “Do you regularly maintain your oral and/or perioral piercings?” In response to the question, “Do you have oral and/or perioral piercings in the following area(s)?” The lips were the favored option for the majority of participants (28%), followed by the uvula (18%), and the nose and tongue (both 18%) (17%). The chin and eyebrows were rarely preferred by the participants (1.1%). In response to the question, “Do you have any intentions to add more oral and/or perioral piercings?” just twenty % of respondents expressed a desire for additional piercings. 11% of the 20% who wanted additional piercings picked the earlobe, 6% liked the belly button, and 3% preferred the ear cartilage.

The study revealed a statistically significant difference (*p* 0.009) between the period since the installation of piercings and the beauty parlor as their source of advice in the event of a complication. This difference was particularly evident in participants who had had piercings for 2 years or less. Conversely, analysis of the link between the interval since the installation of piercings and the intention to undergo an additional piercing revealed a statistically significant difference (*p* 0.043) and that 88% of participants who have had piercings for 2 years or less stated that they do not intend to place additional piercings on this occasion. Nevertheless, there was a statistically significant link (*p* 0.012) between the desire to have new piercings in the same group (piercing 2 years or less) and the period after the installation of the piercings, as expressed by those who preferred to place them in the nose.

**Figure 1:**
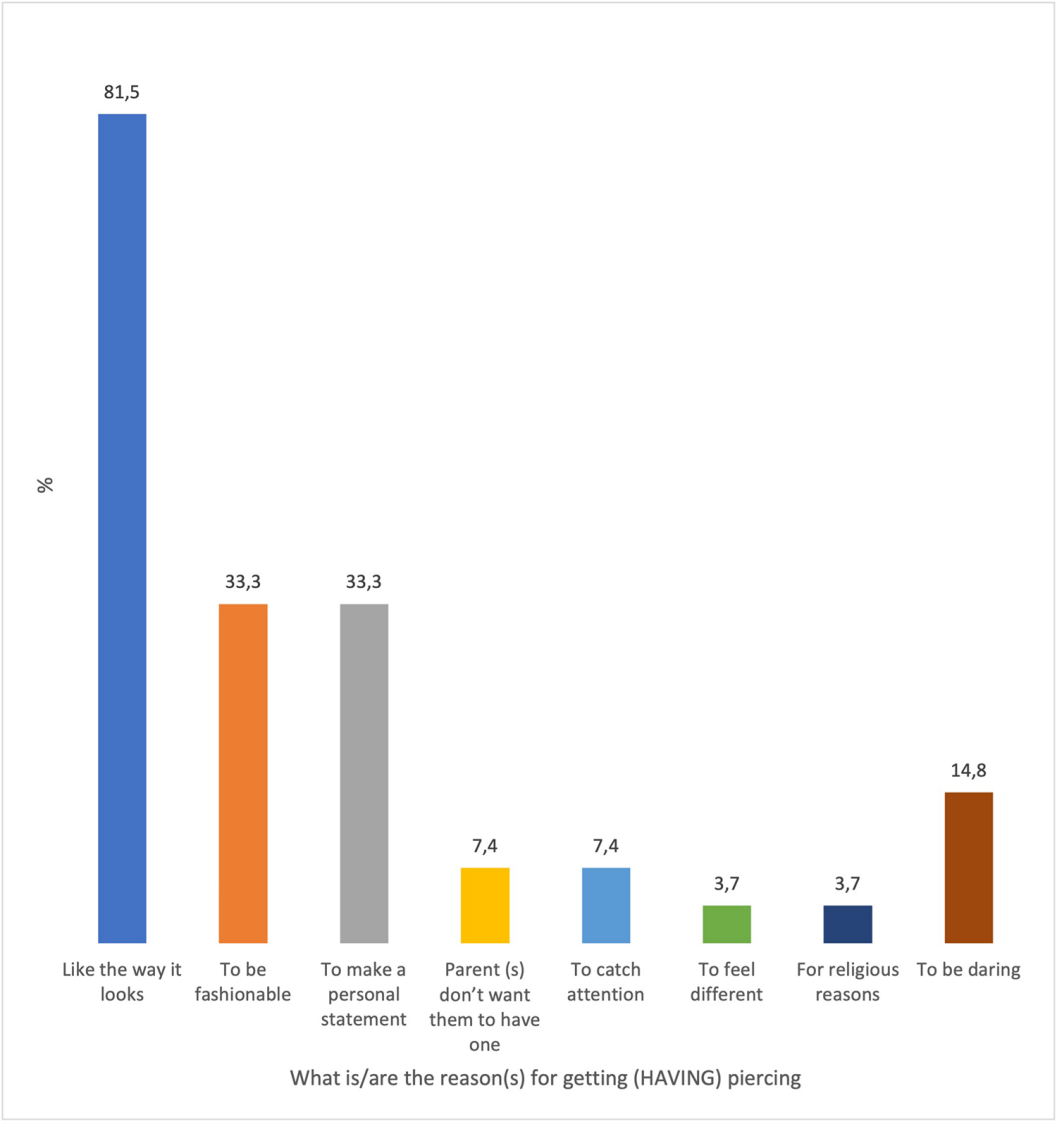
Reasons for oral and/or perioral piercings based on the participants’ opinion. “Participants could choose more than one response; therefore, percentages may exceed%. 100%.”

In response to the question, “Do you have friends who have oral and/or perioral piercings?” Sixty-six % of respondents responded affirmatively. What is(are) the reason(s) for oral and/or perioral piercings in response to the question? Most responders (82%) expressed satisfaction with the item's appearance. Only 33% of respondents desired to be fashionable or make a statement (see Figure 1).

**Figure 2:**
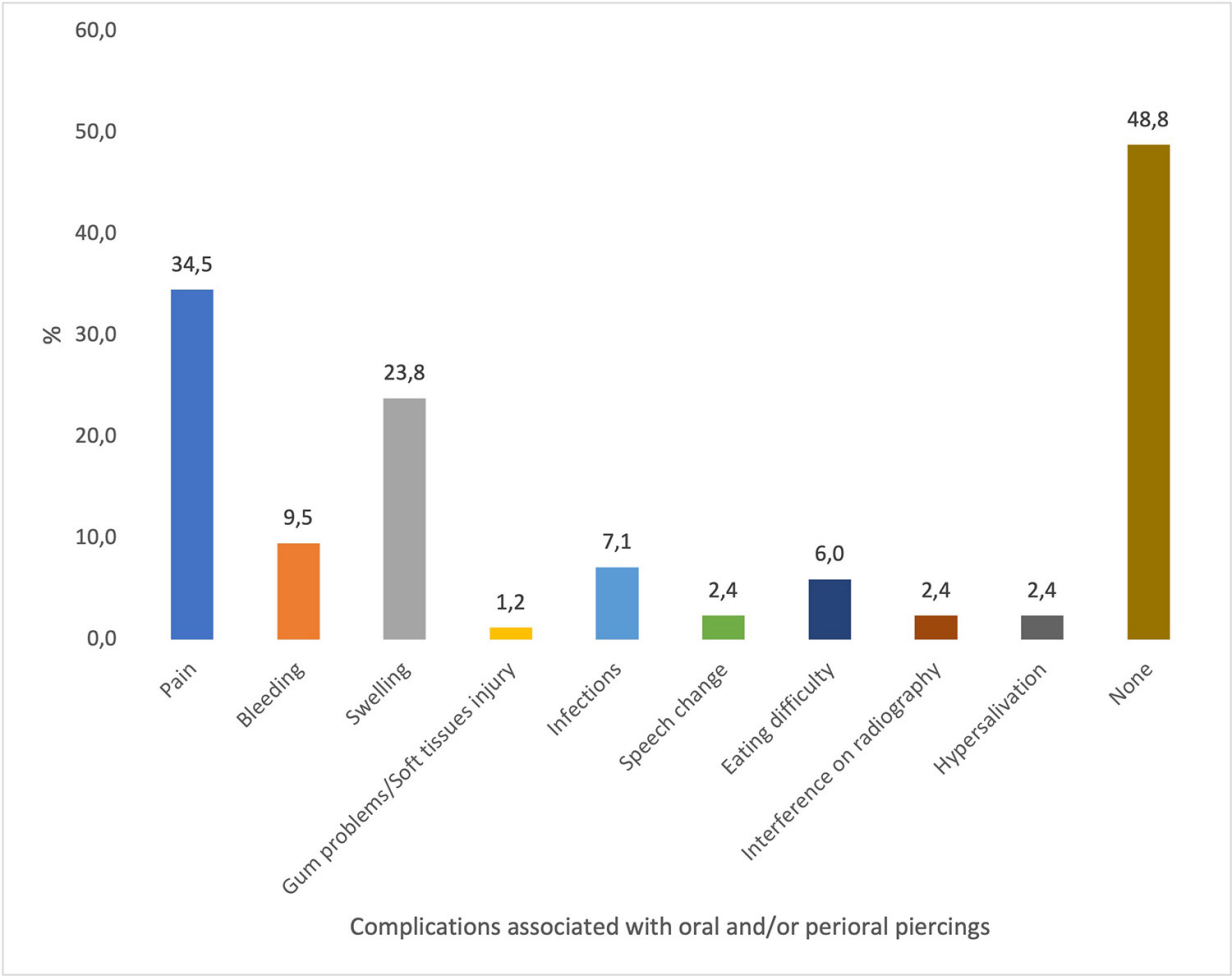
Complications associated with oral and/or perioral piercings.

In answer to difficulties related to oral and/or perioral piercings, the majority (48%) claimed no complications. Nevertheless, 35% of participants who encountered problems reported experiencing pain, while 24% reported experiencing swellings (Figure 2).

In the event of oral and/or perioral piercing issues, the majority of participants (75%) indicated that they would seek the assistance of a beauty parlor. Conversely, just 6% indicated that they would seek assistance from a dentist (Table 1).

**Table 1:** Source of help in case of oral and/perioral piercing complications.

Source of help in case of oral and/perioral piercing complications	No		Yes	
	*N*	%	*N*	%
Source of help				
Doctor	51	70.8	21	29.2
Dentist	68	94.4	4	5.6
Nurse	60	83.3	12	16.7
Beauty parlor	18	25.0	54	75.0
Other, (specify)	72	98.6	1	1.4

Participants could select more than one source; therefore, percentages may exceed 100%.

**Figure 3:**
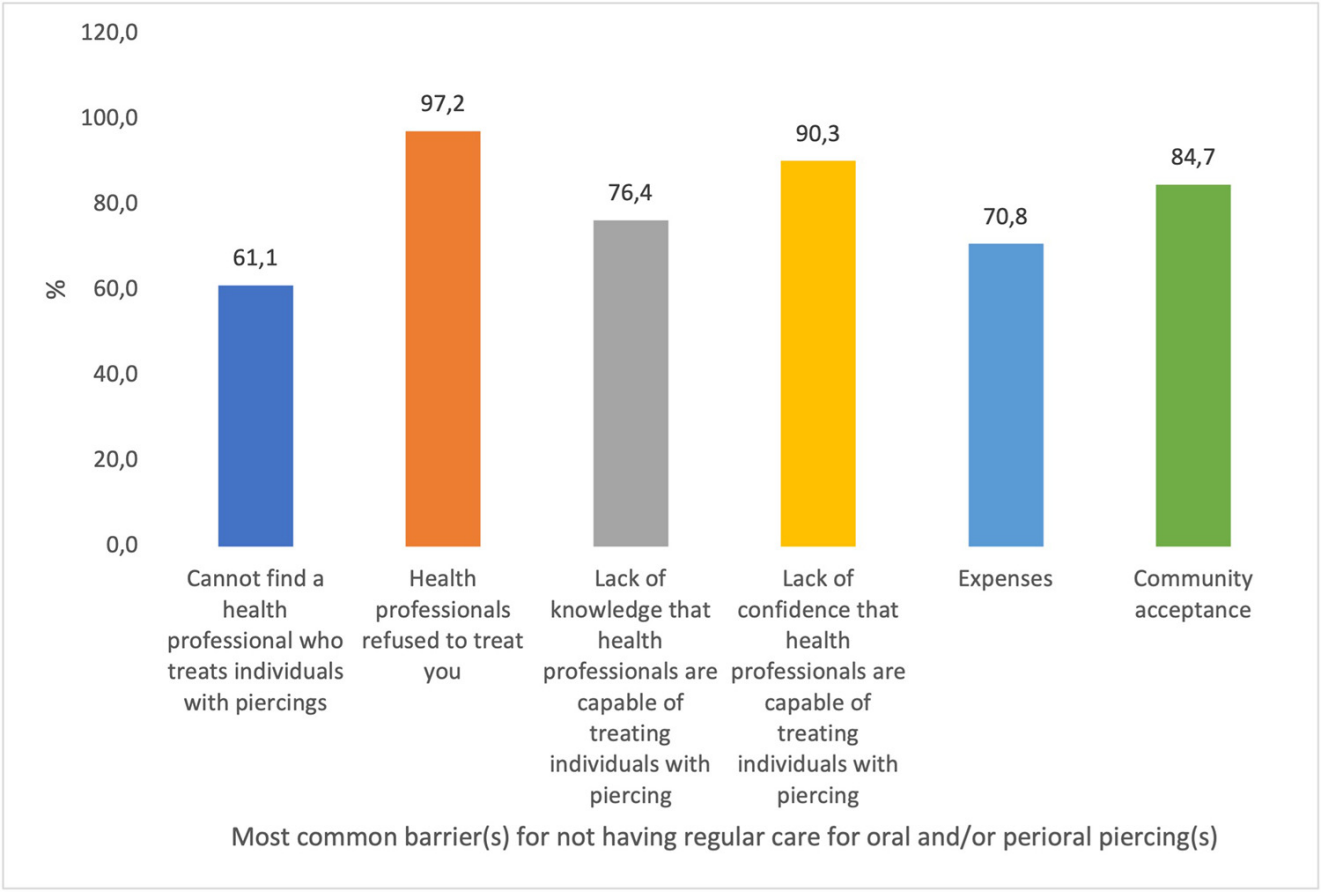
Most common barrier(s) for not having regular care for oral and/or perioral piercing(s). “Participants could choose more than one response; therefore, percentages may exceed 100%.”

The notion that health professionals refuse to treat (97%) and their lack of confidence in their ability to manage the problems were the most prevalent barriers to regular care for oral and/or perioral piercings from the participant's perspective (90%) (see Figure 3).

The oral health behavior and knowledge of participants with oral and/or perioral piercing are detailed in Tables 2, 3.

**Table 2:** Oral health behavior for individuals with oral and/or perioral piercing(s).

Oral health behavior for individuals with oral and/or perioral piercing(s)	*N*	%
How often do you brush your teeth?		
Brushing at least twice a day	42	47.2
Brushing once a day	38	42.7
Seldom or no brush	9	10.1
Time spent tooth brushing		
Less than a minute	27	30.7
More than a minute but less than 2 min	60	68.2
More than 2 min	1	1.1
How often do you use mouth rinse		
Once a day or more	37	48.7
Rarely or none	39	51.3

**Table 3:** Oral health knowledge for individuals with oral and/or perioral piercing(s).

Oral health knowledge for individuals with oral and/or perioral piercing(s)	No		Yes		Don't Know	
	*N*	%	*N*	%	*N*	%
The bacteria mainly cause dental caries	12	13.5	61	68.5	16	18.0
Having sugars can lead to dental caries, or do sweets affect dental health?	7	8.1	74	86.0	5	5.8
Is there any relationship between oral health and overall health?	20	22.7	58	65.9	10	11.4
Is it usual for your gum to bleed while brushing?	65	73.0	19	21	5	6
Is it usual for your gum to be red	52	61.2	22	25.9	11	12.9
Is it usual for your gum to be swollen	68	77.3	11	12.5	9	10.2
Regular brushing protects your teeth	14	15.9	71	80.7	3	3.4
Do you visit a dentist only when you have a toothache?	56	65.1	26	30.2	4	4.7
Do You need a hard toothbrush to clean your teeth?	42	47.2	26	29.2	21	23.6
Is dental floss necessary to keep your teeth clean?	15	16.9	56	62.9	18	20.2

The study revealed a statistically significant difference (*p* 0.018) between the oral and general health group and marital status. Those who were married were more aware of this significance.

The opinion on a correlation between dental health and overall health was significantly varied among the various age groups of respondents (*p* 0.014). This view increased significantly with age, reaching a maximum of 100% in the 31–35 age group. Nevertheless, the percentages were 66.7% and 60% in the 26–30 and over 36 age groups, respectively.

The survey also showed a statistically significant difference (*p* 0.042) among respondents' age groups when asked whether regular tooth brushing benefits tooth protection. This view increased significantly with age, reaching a maximum of 100% in the 31–35 and over-36 age groups. Nevertheless, the 2,630 age group's %age was only 66.7%.

Moreover, the respondents' view that a hard toothbrush is unnecessary to clean their teeth significantly differed among the various age groups (*p* 0.009). This view increased significantly with age, reaching 85% and 90% in the under-15 and 31–35 age groups, respectively. Nevertheless, there was a decrease in the 16–20 age group (41%) and the 26–30 age group (56%).

## Discussion

Body piercing was a cultural activity associated with traditional or religious rites in ancient times. Teenagers and adults are gradually using it as a means of self-expression. Today, it's common to see individuals with oral and/or perioral piercings, which can lead to complications. In addition to poor periodontal and dental health, individuals who have oral and/or perioral piercings sometimes exhibit a variety of problems and side effects. Even healthcare professionals frequently lack an adequate perception of the hazards and difficulties that may develop following the adornment of oral and/or perioral piercings, and the general population is also often ill-informed about these risks and how to minimize them (12–15). The current study is designed to raise awareness of the discrepancy between the literature's verification of the difficulties associated with oral piercings and the knowledge and conduct of persons who have or insert such piercings (14).

Most participants in the present study were between the ages of 22 and 26 and held a university degree. The significant relationship between different age groups of participants and their oral health knowledge indicates that individuals with different age groups perceive a relationship between oral health and overall health. The highest (100%) perception was in the 31–35 age group, contrasting with Reshma et al.'s results, which showed 74.1% that most oral piercings involved teenagers (15).

An Indian study demonstrated that oral and/or perioral piercings appear to be a fairly popular trend; however, the study deduced that accurate statistics of their global prevalence are unknown. Several studies have attempted to deduce data on the global frequency of piercings, but the degree of training, customs, traditions, and social standing significantly affect how common these piercings are (5, 9, 13–17).

Hennequin-Hoenderdos et al. suggest that the majority of oral piercings go well. However, because of the serious short- and long-term consequences (18), oral piercings cannot be recommended (19). It is essential to understand the pervasiveness of oral and/or perioral piercings to determine their impact on the daily practice of dental care professionals. The prevalence of piercings in individuals varies, and the suggestions of a wide range can be attributed to various factors, such as the time and location of the study, differences in the definition of a piercing, and participant categories (20).

The current study demonstrated that oral and/or perioral piercings were predominantly among female participants in the study population. We acknowledge that the gender distribution was skewed in our sample, with 98% of participant being female. This likely reflects cultural and religious norms in Jordan that contribute to the low prevalence of oral and/perioral piercings among men, and unwillingness of them to participate in such studies. This result was mirrored by the study of Aldulaijan et al. The study by Aldulaijan et al. demonstrated an average of 5.2% for oral piercings, with a higher prevalence in females. It has been demonstrated that the most frequently pierced sites are the tongue with a barbell (5.6%), the lips with a labret or ring (1.5%), and the cheek piercings present in (0.1%) of individuals. In contrast, in the current study, the most frequently pierced sites were the lips (28%), followed by the uvula (18%) and the nose and tongue (17%) (14), and a study on college students revealed tongue piercing was the most common piercing (10.4%) (21). A study by Hennequin-Hoenderdos et al. suggested that uvula piercings were less common, attributable to the inherent difficulties in performing the procedure and the prospect of nausea, sore throat, and/or dysphagia (5).

The prevalence of bacterial infections associated with tongue piercings has been inadequately investigated. However, the coincidence between oral piercing and oral bacteria has been frequently established, but other studies have not reached the same conclusion. The prevalence of tongue piercings is not known with precision. In a study of college students, 47 out of 454 respondents (10.4%) reported having their tongues pierced (22). Similarly, the incidence of bacterial infections linked to tongue piercing is also unknown. In an American study, Boardman and Smith discovered that three (5.8%) of 51 respondents with tongue piercings developed an infection, while only two (3.9%) of 51 respondents sought medical or dental attention for these infections (23). Another American study (24) of undergraduate university students revealed that none of the 47 respondents with tongue piercings reported bacterial infections due to the piercing. A survey of tongue-piercing patients in the United Kingdom yielded no reports of bacterial infections among the 122 respondents (25).

Ziebolz et al. conducted microbiological examinations of tongue piercing sites, revealing periodontopathogenic bacteria in jewelry. Additionally, the authors asserted that the periodontopathogenic potential of bacteria changed from moderate to high while the piercing remained in place, and the oral and piercing cleanliness deteriorated (26).

Kapferer et al. acknowledged that disproportionate smoking also appeared to have an impact and that the piercing material contributed to plaque buildup (27). These results underscore the significance of informing patients with oral piercings about the elevated likelihood of contracting bacterial infections. By employing the necessary products to clean and disinfect their jewelry, dentists may help patients with oral piercings preserve the health of their teeth (23).

Due to the potential for several problems, both systemic and oral, the majority of oral healthcare professionals have stated disapproval of their use. Patients commonly are heedless of these outcomes or misjudge them. Therefore, oral healthcare professionals must educate and prepare them to identify and avoid them. The study demonstrated that most participants had piercings inserted in a beauty parlor rather than by a dentist (28). Marketing and advertising related to piercings often focus on beauty salons and barbershops rather than medical or dental clinics. This emphasis reinforces the public perception that beauty parlors are the “normal” or expected place to seek such services.

The significant relationship between the time since the installation of piercings and the beauty parlor as their source of counsel in case of a complication suggests that people with oral piercing for a more extended period might be less likely to seek help from the parlor in case of a complication, which reflects the increased level of self-confidence in handling the complications on their own. The significant relationship between the period since the installation of piercings and the intention to get more piercings suggests that individuals who had piercings for a more extended period are more likely to have another piercing, indicating comfort or interest in body piercing over time. In addition, people who intend to get additional piercings are more likely to get nasal piercings.

Studies by Sahu et al. (17) and Aburaisi et al. (29) demonstrated that 99.1% and 96.9%, respectively, that dentists had seen or treated individuals with oral and/or perioral piercing and in both studies, the dentists indicated tongue piercings as the most prevalent type, followed by lip piercings. A similar result was also demonstrated in the present study. The study by Sahu et al. demonstrated that (77.5%) of their respondents have had complications directly attributable to their piercings (17). In contrast, the study by Aburaisi et al. (79%) showed that dentists who met individuals with piercings offered concerns concerning viable complications of the oral cavity, which included information on the dangers of possible infections, the risk of traumatic destruction to the teeth and gums, and oral hygiene instructions associated with removing the piercings (29).

Individuals acquired piercings for four reasons, as per the current study: the majority cited the piercing's aesthetic appeal, the desire to be fashionable, the desire to make a personal statement, and the desire to be daring. Additionally, their parents or friends believed that the piercing was cute, indicating that their parents or friends approved of the piercing. Aburaisi et al. reported a comparable finding (29, 30).

The results of the current study indicate that individuals who had oral piercings were cognizant of the potential dangers and problems that could affect their oral and overall health. The majority of individuals reported experiencing swellings or soreness after the implantation of their piercings. Most participants sought assistance from the beauty salon, while a small number sought advice from dentists regarding piercings, which may be attributed to the emergence of social media or self-awareness. The preference for beauty parlors as a source of care in cases of oral and/or perioral piercing complications may be influenced by several factors. First, there may be a general lack of awareness that medical professionals—such as doctors, dentists, and nurses—are trained and qualified to provide care or advice related to piercings. In contrast, beauty parlors may be perceived as more familiar or accessible environments for such services. Additionally, oral and perioral piercings remain a relatively recent trend in Jordanian society, particularly among youth. As a result, individuals may follow social norms or peer behavior rather than seek professional medical guidance. These factors combined may contribute to the higher reliance on beauty parlors as the primary source of care in piercing-related cases.

Furthermore, gingival recession was observed in a few instances following perioral lip piercing (6, 18, 19, 26).

In the present study, the most common barrier perceived by the participants was the refusal of treatment by oral health professionals. Also, the perceived barrier by the participants was the lack of confidence in the oral health profession and their lack of knowledge of piercings. Therefore, to encourage patients to discuss their oral health concerns and piercing difficulties, research and professional education are required for pre-graduation of medical and dental students, and health professionals need to be informed of the risks and barriers to health care linked to oral piercings (27). Salama et al. mirrored this point of view and concluded that health professionals are concerned about oral and/or perioral piercing, yet it's still common. They further concluded that they often witness the difficulties it can lead to. Research and professional education are required to help health professionals advise and protect patients (28, 31).

Regarding oral health knowledge for individuals with oral and/or perioral piercing(s), most participants knew that sugars and sweets negatively impact dental health and induce tooth caries and that regular tooth brushing kept their teeth safe. Concurrently, many participants were aware that bacteria caused tooth caries. Also, many participants knew that dental and overall health were related. In contrast, many participants believed that swollen gums were normal. These findings have been mirrored by the study of Ridout, which concluded that several oral health conditions, such as caries, plaque buildup, gingivitis/periodontitis, dental fractures, bleeding, inflammation, and swollen gums, have been related to oral piercings (4).

The current study corroborates the findings in the literature, which demonstrate that complications and knowledge of oral health of oral and/or perioral piercings include allergic responses, infection, inflammation of the gingiva, tooth chipping, and halitosis. Additionally, this study confirms the prior literature's support that someone with the necessary professional training should do oral piercings.

The significance difference in oral and general health awareness between married and unmarried participants might be explained by the fact that married individuals may be more maintain good health for the well-being of their family, leading them to adopt better oral care practices. Additionally, marriage may promote health awareness, as partners often encourage and remind each other about maintaining healthy practices, including oral health. The significance difference in awareness regarding the benefits of regular tooth brushing across different age groups. Older respondents demonstrated a significantly higher level of knowledge compared to younger participants. This may be explained due to the accumulated knowledge, more experience with oral health issues, and increased opportunities to receive oral health information that assist in a better understanding of preventive practices. The age-related differences in awareness between age groups suggest that oral health education could be more targeted to younger individuals to improve their understanding and promote healthier behaviors. Additionally, significant differences were found in respondents' views regarding the necessity of using a hard toothbrush to clean teeth, this view increased with age. This could reflect varying levels of education and experience with dental care products. Due to different dental cultural beliefs, and generational differences in dental care.

## Conclusion

The findings of this study highlight significant gaps in oral health knowledge and behavior among individuals with oral and/or perioral piercings, particularly among younger and unmarried participants. These gaps may increase the risk of oral complications and contribute to delays in seeking professional dental care. The high reliance on non-medical sources, such as beauty parlors, for piercing-related issues suggests a need for public health interventions aimed at raising awareness about the importance of seeking care from qualified healthcare providers. Furthermore, the gender imbalance observed may reflect underlying cultural norms, but also points to an unmet need for research on male populations. Also, highlights the lack of data on male individuals in Jordanian male. Targeted health education campaigns should be sensitive to cultural factors while promoting inclusive access to oral health services.

### Recommendations

Additional study in this field is required, with a more diversified population and a greater number of participants. Public health authorities should initiate and sustain awareness efforts due to the public's inadequate comprehension of these issues. Dental professionals should be trained to provide non-judgmental, culturally competent counseling on oral piercings, including proper hygiene, risks, and when to seek care. Public health efforts could also involve partnerships with beauty parlors and social influencers to disseminate accurate oral health information to youth.

This study is among the first in Jordan to specifically investigate oral health knowledge, behavior, and barriers to dental care among individuals with oral and/or perioral piercings filling a gap in the existing literature. In addition, the study considers cultural and religious factors that may affect prevalence of piercings and health-seeking behavior, adding depth and relevance to the findings. Furthermore, the result of this study reveals significant gaps in awareness and care-seeking behavior, which can help guide future oral health education initives and outreach campaigns for specific groups.

Future studies should aim to recruit more representative samples using probability-based methods to improve generalizability. Additionally, qualitative research could provide deeper insight into motivations, beliefs, and barriers behind piercing-related health behaviors in different demographic groups. Studies comparing knowledge and outcomes between individuals who seek care from medical professionals vs. beauty parlors would also be valuable.

### Limitations

This study has several important limitations that may affect the generalizability of the results. The results of this investigation are the result of a cross-sectional survey that is considered to be representative and diverse. However, selection bias likely influenced the results, as most studies utilized convenience samples to survey participants. Additionally, the sample size was small, which is one of the drawbacks of the current study although slightly exceeding the calculated, limits the statistical power to detect subtle differences between subgroups. In addition, most participants in this study were female, which may reflect societal norms in Jordan, where piercing are more commonly practiced among females than males. Imbalance in gender distribution is considered one of the biggest limitations of the study, as a results, the findings might not be representative of the Jordanian men with oral and/or perioral piercings. It is challenging to extrapolate the characteristics of this investigation to other groups.

There are potential additional limitations, such as inadequate awareness of the tooth wear in the vicinity of the piercing sites, dental hygiene practices, the duration of the piercing, and the stem length of the piercing instruments.

However, this investigation provides some insight into the correlation between oral and/or perioral piercings. Furthermore, the present study employed a questionnaire to gather data, as with most studies investigating oral health knowledge and barriers or behaviour of piercings. Therefore, the probability of information biases and selection must be considered. Therefore, it is essential to use caution when attempting to conclude the findings of the present investigation.

## Data Availability

The original contributions presented in the study are included in the article/Supplementary Material, further inquiries can be directed to the corresponding authors.
